# A Wide-Swath Spaceborne TOPS SAR Image Formation Algorithm Based on Chirp Scaling and Chirp-Z Transform

**DOI:** 10.3390/s16122095

**Published:** 2016-12-09

**Authors:** Wei Yang, Jie Chen, Hong Cheng Zeng, Peng Bo Wang, Wei Liu

**Affiliations:** 1 School of Electronic and Information Engineering, Beihang University, Beijing 100191, China; yangweigigi@buaa.edu.cn (W.Y.); zenghongcheng@buaa.edu.cn (H.C.Z.); wangpb7966@buaa.edu.cn (P.B.W.); 2 Collaborative Innovation Center of Geospatial Technology, Wuhan 430079, China; 3 Electronic and Electronic Engineering Department, University of Sheffield, Sheffield S1-3JD, UK; w.liu@sheffield.ac.uk

**Keywords:** SAR, TOPS, modified azimuth deramp, chirp-z

## Abstract

Based on the terrain observation by progressive scans (TOPS) mode, an efficient full-aperture image formation algorithm for focusing wide-swath spaceborne TOPS data is proposed. First, to overcome the Doppler frequency spectrum aliasing caused by azimuth antenna steering, the range-independent derotation operation is adopted, and the signal properties after derotation are derived in detail. Then, the azimuth deramp operation is performed to resolve image folding in azimuth. The traditional dermap function will introduce a time shift, resulting in appearance of ghost targets and azimuth resolution reduction at the scene edge, especially in the wide-swath coverage case. To avoid this, a novel solution is provided using a modified range-dependent deramp function combined with the chirp-z transform. Moreover, range scaling and azimuth scaling are performed to provide the same azimuth and range sampling interval for all sub-swaths, instead of the interpolation operation for the sub-swath image mosaic. Simulation results are provided to validate the proposed algorithm.

## 1. Introduction

State-of-the-art spaceborne SAR systems are capable of operating in several novel imaging modes by adopting the electronic beam steering technique in both elevation and along-track directions, and one such an example is the terrain observation with progressive scan (TOPS) mode [1,2,3]. The TOPS mode was presented by Zan and Guarnieri in [1] for wide-swath observation, similar to the scanSAR mode [3]. Compared with the scanSAR mode, the TOPS mode can avoid scalloping and the azimuth-dependent distributed target ambiguity ratio effect on SAR images, improving the image radiometric quality significantly [3]. Therefore, the TOPS mode is not only widely used in current spaceborne SAR systems, such as Sentinel-1 and TerraSAR-X/TanDem-X [4,5,6], but also adopted in future SAR systems, such as TerraSAR-X2 [7].

The TOPS mode scans the scene with very long bursts and rotates the antenna beam throughout the acquisition from backward to forward in azimuth, resulting in a wider total Doppler bandwidth compared with the pulse repetition frequency (PRF) and a longer strip compared with the standard strip mode within the same time span [1], so classic strip algorithms are not suitable for processing the TOPS data due to Doppler frequency spectrum aliasing and image folding in azimuth.

Several algorithms were developed to overcome these problems. The wavenumber domain algorithm (WDA) kernel was first adopted for TOPS data processing by adding pre- and post- processing steps in [1,8]. However, the WDA cannot accommodate the effective velocity variation along the slant range, so it is not suitable for wide-swath coverage TOPS data processing. In order to improve processing efficiency, the extended chirp scaling algorithm (ECSA) was employed for TerraSAR-X TOPS data processing [9]. Based on the ECSA, a more efficient algorithm, called the baseband azimuth scaling algorithm (BASA) [10,11], was proposed using the azimuth scaling technique. However, both ECSA and BASA are sub-aperture algorithms, and a raw data division operation is required to overcome Doppler frequency spectrum aliasing. Therefore, sub-aperture recombination has to be performed during data processing. In addition to the sub-aperture algorithms, full-aperture algorithms were also proposed, such as the three-step algorithm [12,13], which employ the derotation technique and the deramp technique to avoid the Doppler frequency spectrum aliasing problem and image folding, respectively. However, the deramp operation will introduce a time shift [14], resulting in the appearance of ghost targets and azimuth resolution reduction at the scene edge, especially in the wide-swath coverage case. In order to accommodate the time shift, a modified de-rotation factor was introduced in [15,16], at the expense of Doppler frequency spectrum extension during the de-rotation operation, which requires an increased PRF and leads to limitations on mode design [4,5,17]. Moreover, in order to satisfy application requirements, such as interferometric and monitoring applications [18,19,20], a large coverage area is needed by increasing the antenna steering span [7], and adopting the azimuth multiple-channel technique [21]. Consequently, an efficient and accurate wide-swath coverage TOPS image formation algorithm is required.

In this paper, an efficient and accurate TOPS image formation algorithm is proposed for wide-swath coverage TOPS data focusing without raw data division. In the proposed algorithm, the range-independent de-rotation operation is employed to overcome the spectrum aliasing problem, and the signal properties after de-rotation are derived in detail. Moreover, a modified range-dependent deramp function is proposed to accommodate the time shift, and the chirp-z transform is utilized for correcting geometry distortions caused by the range-dependent deramp operation. Furthermore, range scaling and azimuth scaling are performed, providing the same pixel sampling interval for all sub-swath images in both range and azimuth dimensions respectively, which avoids interpolation for the sub-swath image mosaic. Simulation results are provided to demonstrate the performance of the proposed algorithm.

This paper is organized as follows: the signal model for the TOPS mode is provided in Section 2 and the imaging algorithm is proposed in Section 3. Simulation results are presented in Section 4, with conclusions drawn in Section 5.

## 2. Signal Model of TOPS Mode

### 2.1. Acquisition Geometry

**Figure 1:**
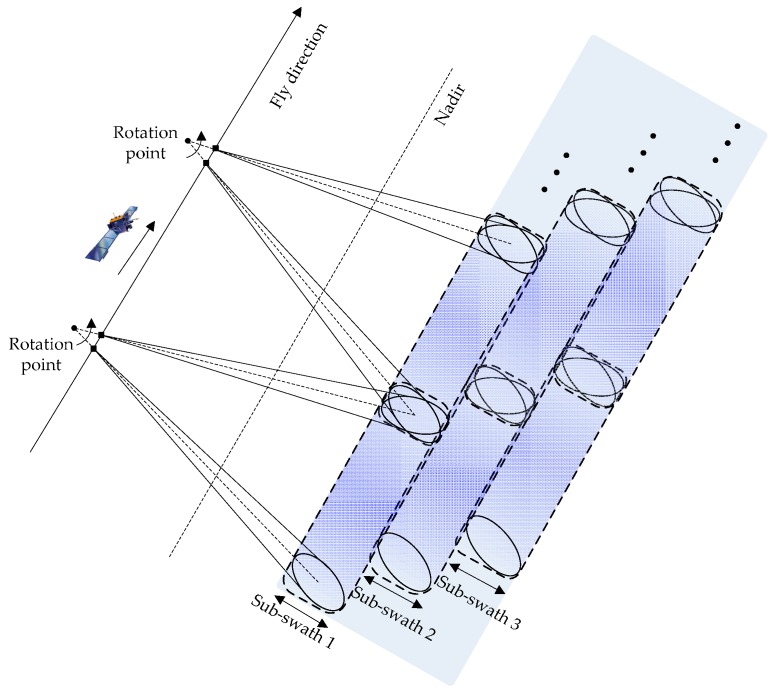
Acquisition geometry of the spaceborne TOPS mode.

In conventional ScanSAR mode, targets located at different azimuth positions are illuminated by different parts of the azimuth antenna pattern (AAP), resulting in problems of scalloping, and azimuth-varying distributed target ambiguity ratio and signal-to-noise ratio (SNR). To overcome these problems, the antenna steering technique is employed in the TOPS mode. The azimuth antenna beam is rotated throughout the acquisition from backward to forward, acquiring very long bursts. After finishing the scan of the sub-swath 1, the look angle is changed to illuminate the sub-swath 2, performing the scan from backward to forward again. When the last sub-swath is illuminated, the antenna direction moves back to the sub-swath 1, as shown in Figure 1. Consequently, all the targets are weighted by the complete AAP, which eliminates the scalloping, azimuth-varying ambiguity and SNR.

**Figure 2:**
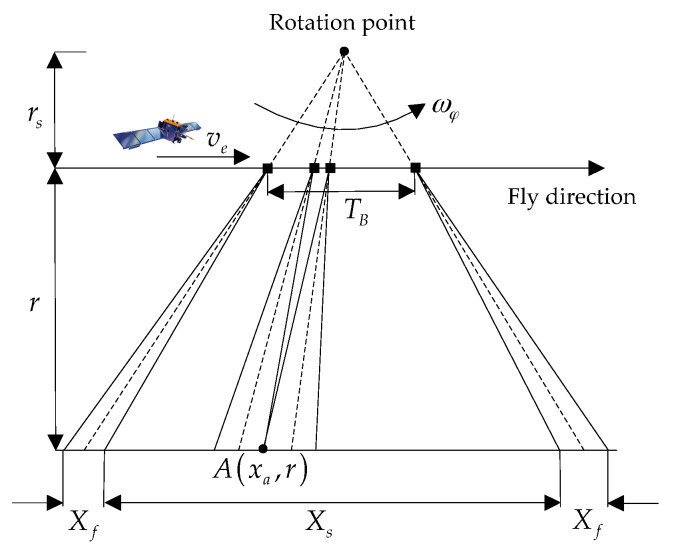
Acquisition geometry of the TOPS mode in the slant range plane.

The acquisition geometry of the TOPS mode in the slant range plane is shown in Figure 2, where $r_{s}$ and r represent the minimum distance from the virtual rotation point to the SAR sensor and to the ground, respectively, $v_{e}$ is the effective velocity, $T_{B}$ is the burst time, A is the point target located at ($x_{a},r$), $X_{s}$ corresponds to the valid area with full illumination time, and $X_{f}$ corresponds to the area with insufficient illumination time. The TOPS mode employs a counter-clockwise antenna beam steering at a constant rotation rate of $\omega_{\varphi }$, leading to reduction of target illumination time. By adopting the hybrid factor γ(r) [1], the azimuth resolution $\rho_{a}$ can be approximately given by:

(1) $$\rho_{a}\approx \frac{D}{2\cdot \gamma \left(r\right)}$$

where D is the antenna length, and γ(r) is:

(2) $$\gamma \left(r\right)=\frac{r_{s}}{r_{s}+r}=\frac{1}{1+r/r_{s}}$$

According to Equations (1) and (2), the azimuth resolution is dependent on r, which means that targets located at the near slant range can have a higher azimuth resolution than those located at the far slant range.

As shown in Figure 2, assuming a linear frequency modulated (LFM) pulse is transmitted by the radar, after demodulation to the baseband, the received signal for point target A can be described as [13]:

(3) $$\begin{array}{ll} S\left(\tau ,\eta ;x_{a},r\right) & =rect\left[\tau -\frac{2r\left(\eta ;x_{a},r\right)}{c}\right]\cdot rect\left[\frac{v_{e}\eta /\gamma \left(r\right)-x_{a}}{X_{f}}\right]\cdot rect\left[\frac{\eta }{T_{B}}\right]\cdot rect\left[\frac{x_{a}}{X_{s}}\right]\\ & \cdot \text{exp}\left\{-j\pi b{\left(\tau -\frac{2R\left(\eta ;x_{a},r\right)}{c}\right)}^{2}\right\}\cdot \text{exp}\left\{-j\frac{4\pi R\left(\eta ;x_{a},r\right)}{\lambda }\right\} \end{array}$$

where rect[⋅] represents the rectangular window, *b* is the signal frequency modulation rate, *c* is the speed of light, λ is the carrier frequency, τ and η are range time and azimuth time, respectively, $X_{f}=\lambda r/D$ is the length of azimuth antenna beam footprint, and $R\left(\eta ;x_{a},r\right)$ is the distance between the satellite and the point target $A\left(x_{a},r\right)$. Defining $x_{a}=v_{e}\eta_{a}$, $R\left(\eta ;x_{a},r\right)$ can be expressed as follows:

(4) $$R\left(\eta ;\eta_{a},r\right)=\sqrt{r^{2}+v_{e}^{2}{\left(\eta -\eta_{a}\right)}^{2}}.$$

### 2.2. Signal Model

TOPS is proposed as a wide-swath imaging mode with low/medium resolutions. So, the range cell migration (RCM) is relatively small, which can be corrected by classic algorithms, such as the chirp scaling algorithm (CSA) [22], and the Range-Doppler algorithm (RDA) [23]. After range cell migration correction (RCMC), the azimuth signal expression is given by:

(5) $$S_{A,\eta }\left(\eta ;\eta_{a},r\right)=rect\left[\frac{v_{e}\eta /\gamma \left(r\right)-v_{e}\eta_{a}}{X_{f}}\right]\cdot rect\left[\frac{\eta }{T_{B}}\right]\cdot rect\left[\frac{v_{e}\eta_{a}}{X_{s}}\right]\cdot \text{exp}\left\{-j\pi k_{s}\left(r\right){\left(\eta -\eta_{a}\right)}^{2}\right\}$$

Applying the azimuth fast Fourier transform (FFT) to Equation (5), the azimuth signal Doppler spectrum is:

(6) $$S_{A,f_{\eta }}\left(f_{\eta };\eta_{a},r\right)=rect\left[-\frac{f_{\eta }-k_{s}\left(r\right)\left(1-\gamma \left(r\right)\right)\eta_{a}}{B_{\Delta \theta }\gamma \left(r\right)}\right]\cdot rect\left[-\frac{f_{\eta }-k_{s}\left(r\right)\eta_{a}}{k_{s}\left(r\right)T_{B}}\right]\cdot rect\left[\frac{v_{e}\eta_{a}}{X_{s}}\right]\cdot \text{exp}\left\{j\pi \frac{f_{\eta }^{2}}{k_{s}\left(r\right)}-2\pi f_{\eta }\eta_{a}\right\}$$

where $k_{s}\left(r\right)$ is the Doppler rate, and $B_{\Delta \theta }$ is the Doppler bandwidth corresponding to the instantaneous antenna beamwidth, as given below:

(7) $$k_{s}\left(r\right)=\frac{2v_{e}^{2}}{\lambda r}$$

(8) $$B_{\Delta \theta }=\frac{2v_{e}}{D}$$

**Figure 3:**
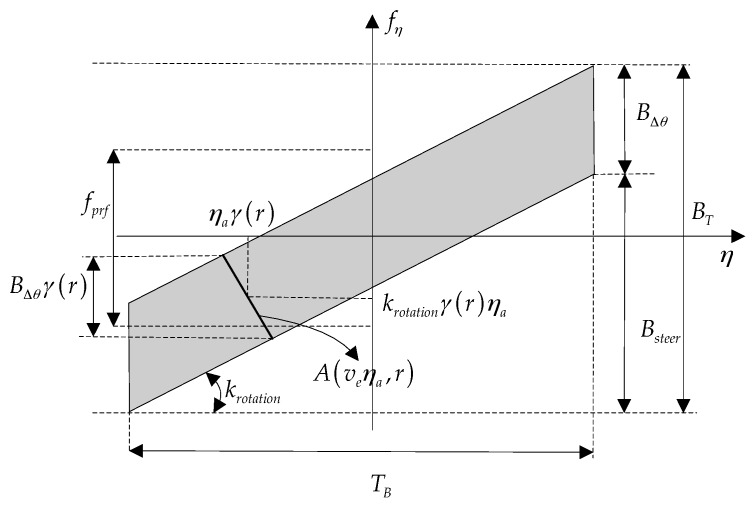
Azimuth time-frequency diagram of TOPS mode.

The properties of azimuth signal Doppler spectrum have been analyzed in detail in [1]. As the time-frequency diagram (TFD) shown in Figure 3, the total azimuth bandwidth $B_{T}$, consisting of $B_{\Delta \theta }$ and $B_{steer}$, may span over serval PRF intervals that indicated by $f_{prf}$, which results in Doppler frequency spectrum aliasing. $B_{steer}$ denotes the Doppler bandwidth resulting from azimuth antenna-beam steering, given by:

(9) $$B_{steer}=k_{rotation}\cdot T_{B}$$

where $k_{rotation}$ is the sweep rate [6]:

(10) $$k_{rotation}=\frac{2v_{e}^{2}}{\lambda r_{s}}$$

In order to overcome spectrum aliasing, some methods have been proposed, including frequency mosaic [1], sub-aperture processing [10], and the derotation operation [13,15]. Among them the derotation operation is the most efficient. Moreover, the related range-dependent and range-independent functions were derived in [10,13], respectively.

Another problem in TOPS imaging is image folding in azimuth and one solution is the deramp technique. However, the time shift caused by the deramp operation requires an increased PRF [14]. On the other hand, the wide-swath coverage needs a low PRF for a large echo-receiving window, which leads to contradiction between efficient processing and wide-swath coverage. As a solution, in this work, a novel image formation algorithm is proposed for wide-swath coverage TOPS data processing, which can overcome the problems of spectrum aliasing, image folding and time shift without sub-aperture combination.

## 3. Imaging Algorithm

### 3.1. Azimuth De-Rotation

Similar to the case of sliding spotlight [24,25], the range-independent de-rotation function is employed to overcome the Doppler frequency spectrum aliasing problem, with the expression given by:

(11) $$H_{de\_ro}\left(\eta \right)=\text{exp}\left\{-j\pi k_{rotation}\eta^{2}\right\}$$

The de-rotation operation involves azimuth signal convolution between the azimuth signal and de-rotation function. Based on the principle of stationary phase (POSP) [26], the convolution result is given by:

(12) $$\begin{array}{ll} {S^{\prime }}_{A,\eta }\left(\eta ;\eta_{a},r\right) & =S_{A,\eta }\left(\eta ;\eta_{a},r\right)⊗H_{de\_ro}\left(\eta \right)\\ & =rect\left[\frac{\eta }{\lambda r_{s}/\left(Dv_{e}\right)}\right]\cdot rect\left[\frac{\eta_{a}}{X_{s}/v_{e}}\right]\cdot rect\left[\frac{\eta +\eta_{a}r_{s}/r}{T_{B}\left(r+r_{s}\right)/r}\right]\cdot \text{exp}\left\{-j\pi k_{e}\left(r\right){\left(\eta -\eta_{a}\right)}^{2}\right\} \end{array}$$

where:

(13) $$k_{e}\left(r\right)=\frac{2v_{e}^{2}}{\lambda \left(r_{s}+r\right)}$$

In the next, we analyze the value range of the azimuth signal in detail. According to Equation (12), after de-rotation, the value range of the azimuth signal is determined by three terms. The first term limits the value of η by:

(14) $$\eta_{1}\in \left[-\frac{\lambda r_{s}}{2Dv_{e}},\frac{\lambda r_{s}}{2Dv_{e}}\right]$$

With respect to $\eta_{a}$, the value range is determined by the second term:

(15) $$\eta_{a}\in \left[-\frac{X_{s}}{2v_{e}},\frac{X_{s}}{2v_{e}}\right]$$

Considering the third term, the value of η varies with $\eta_{a}$. As a result:

(16) $$\eta_{2}\in \left[-\frac{T_{B}\left(r+r_{s}\right)}{2r}-\eta_{a}\frac{r_{s}}{r},\frac{T_{B}\left(r+r_{s}\right)}{2r}-\eta_{a}\frac{r_{s}}{r}\right]$$

Substituting Equation (15) into Equation (16), we can obtain the value of η corresponding to targets located at the azimuth edge, with $\eta_{a}=X_{s}/(2v_{e})$ and $\eta_{a}=-X_{s}/(2v_{e})$, respectively:

(17) $${\eta_{2}|}_{\eta_{a}=\frac{X_{s}}{2v_{e}}}\in \left[-\frac{T_{B}\left(r+r_{s}\right)}{2r}-\frac{X_{s}}{2v_{e}}\frac{r_{s}}{r},\frac{T_{B}\left(r+r_{s}\right)}{2r}-\frac{X_{s}}{2v_{e}}\frac{r_{s}}{r}\right]$$

(18) $${\eta_{2}|}_{\eta_{a}=-\frac{X_{s}}{2v_{e}}}\in \left[-\frac{T_{B}\left(r+r_{s}\right)}{2r}+\frac{X_{s}}{2v_{e}}\frac{r_{s}}{r},\frac{T_{B}\left(r+r_{s}\right)}{2r}+\frac{X_{s}}{2v_{e}}\frac{r_{s}}{r}\right]$$

Comparing Equations (14) and (17), the boundary value difference can be calculated as follows:

(19) $$\begin{array}{cc} \Delta \eta_{d} & {|}_{\eta_{a}=X_{s}/(2v_{e})}=\frac{\lambda r_{s}}{2Dv_{e}}-\left(\frac{T_{B}\left(r+r_{s}\right)}{2r}-\frac{X_{s}}{2v_{e}}\frac{r_{s}}{r}\right)=\frac{\lambda r_{s}}{2Dv_{e}}+\frac{X_{s}}{2v_{e}}\frac{r_{s}}{r}-\frac{T_{B}\left(r+r_{s}\right)}{2r}\\ & =\frac{X_{f}}{2v_{e}}\frac{r_{s}}{r}+\frac{X_{s}}{2v_{e}}\frac{r_{s}}{r}-\frac{T_{B}\left(r+r_{s}\right)}{2r}=\frac{\left(X_{s}+X_{f}\right)}{v_{e}}\frac{r_{s}}{\left(r+r_{s}\right)}\frac{\left(r+r_{s}\right)}{2r}-\frac{T_{B}\left(r+r_{s}\right)}{2r}\\ & =\frac{T_{B}\left(r+r_{s}\right)}{2r}-\frac{T_{B}\left(r+r_{s}\right)}{2r}=0 \end{array}$$

**Figure 4:**
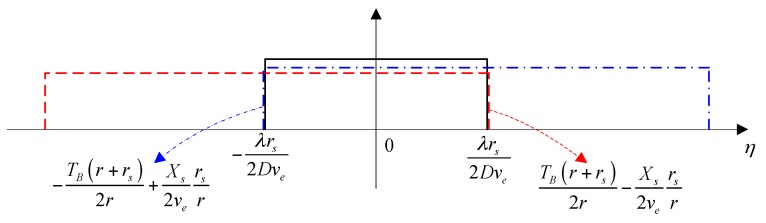
The value range of azimuth signal after a derotation operation, which is limited by Equation (14) (black solid rectangular window), Equation (17) (red dashed rectangular window), and Equation (18) (blue dash-dot rectangular window).

In the same way, we can calculate the boundary value difference with respect to the target located at $\eta_{a}=-X_{s}/(2v_{e})$, which is also equal to zero. As shown in Figure 4, the range of $\eta_{2}$ is always wider than $\eta_{1}$ for all targets located in $X_{s}$. So, the first term plays a dominant role in Equation (12), and the azimuth signal after de-rotation can be rewritten as follows:

(20) $${S^{\prime }}_{A,\eta }\left(\eta ;\eta_{a},r\right)=rect\left[\frac{\eta }{\lambda r_{s}/\left(Dv_{e}\right)}\right]\cdot \text{exp}\left\{-j\pi k_{e}\left(r\right){\left(\eta -\eta_{a}\right)}^{2}\right\}$$

After de-rotation, the equivalent PRF, referred to as ${f^{\prime }}_{prf}$, is given by:

(21) $${f^{\prime }}_{prf}=\frac{N_{1}k_{rotation}}{f_{prf}}$$

where $N_{1}$ is the azimuth output point number after de-rotation. Moreover, ${f^{\prime }}_{prf}$ should be larger than $B_{T}$ to resolve spectrum aliasing, which determines the value of $N_{1}$ by [12]:

(22) $$N_{1}>N_{A}+f_{prf}\frac{\lambda r_{s}}{Dv_{e}}$$

where $N_{A}$ is the azimuth point number of raw data.

With azimuth FFT, ${S^{\prime }}_{A,\eta }\left(\eta ;\eta_{a},r\right)$ is transformed into the azimuth Doppler domain, yielding:

(23) $${S^{\prime }}_{A,f_{\eta }}\left(f_{\eta };\eta_{a},r\right)=rect\left[-\frac{f_{\eta }-k_{e}\left(r\right)\eta_{a}}{B_{\Delta \theta }\gamma \left(r\right)}\right]\cdot \text{exp}\left\{j\pi \frac{f_{\eta }^{2}}{k_{e}\left(r\right)}-j2\pi f_{\eta }\eta_{a}\right\}$$

Since the range-independent derotation operation is performed, the azimuth signal is completely overlapped in the time domain, as shown in Equation (20). Moreover, the time domain width of the output signal, referred to as $T_{DE}$, is wider than the time domain width of the azimuth signal, as shown in Equation (24), which means that the derotation operation will not cause time aliasing:

(24) $$T_{DE}=\frac{f_{prf}}{k_{rotation}}>\frac{B_{\Delta \theta }}{k_{rotation}}=\frac{\lambda r_{s}}{Dv_{e}}$$

Furthermore, according to Equation (23), the frequency domain width after derotation is limited by the first term, so targets located at different azimuth positions are separated, with the accumulated Doppler bandwidth $B_{tar}\left(r\right)$ and Doppler centroid $f_{\eta ,d}\left(r\right)$ given by:

(25) $$B_{tar}\left(r\right)=B_{\Delta \theta }\gamma \left(r\right)$$

(26) $$f_{\eta ,d}\left(r\right)=k_{e}\left(r\right)\eta_{a}$$

**Figure 5:**
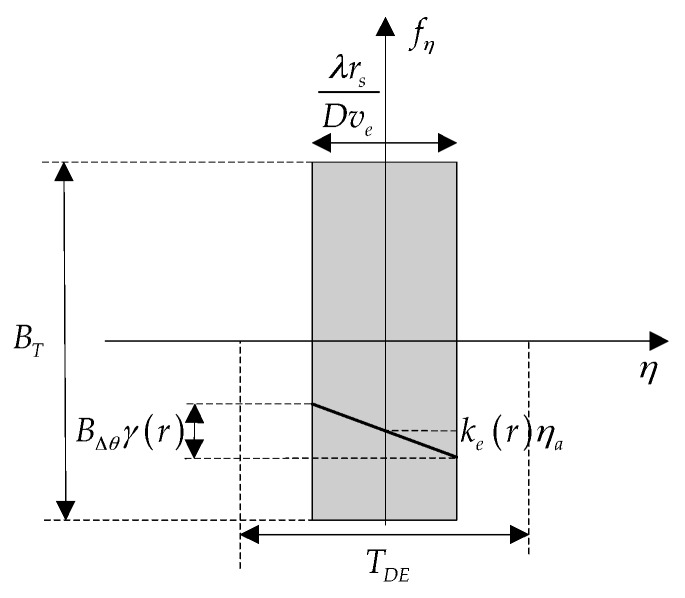
Azimuth time-frequency diagram after derotation.

Note that $B_{tar}\left(r\right)$ and $f_{\eta ,d}\left(r\right)$ are range-dependent, but the total azimuth bandwidth $B_{T}$ is range-independent. Figure 5 shows the azimuth signal time-frequency diagram after the derotation operation.

### 3.2. Range Scaling

The TOPS sub-swaths data have different signal sampling rate $f_{s}$, resulting in different sampling intervals in the range dimension. Consequently, resampling in range is necessary for sub-swaths combination, requiring additional computation. In order to avoid the resampling operation, range scaling is performed in the range-Doppler domain, providing the same sampling intervals. According to the range scaling principle proposed in [14], the range scaling function is given by:

(27) $$H_{scl,r}\left(f_{\eta },\tau \right)=\text{exp}\left\{-j\pi b\left(f_{\eta };r_{ref}\right)Cs^{\left(i\right)}\left(f_{a}\right){\left(\tau -\frac{2R\left(f_{\eta };r_{ref}\right)}{c}\right)}^{2}\right\}$$

where $r_{ref}$ is the reference range, $b\left(f_{a};r_{ref}\right)$ and $2R\left(f_{\eta };r_{ref}\right)/c$ are the effective FM chirp rate in range and the time delay in range-Doppler domain, respectively, whose expression can be found in [22], and $Cs^{\left(i\right)}\left(f_{a}\right)$ is the range scaling factor:

(28) $$Cs^{\left(i\right)}\left(f_{a}\right)=\frac{\alpha^{\left(i\right)}}{\sqrt{1-{\left(\frac{\lambda f}{2v_{e}}\right)}^{2}}}-1$$

where *i* stands for the *i*-th sub-swath, and $\alpha^{\left(i\right)}$ is given by:

(29) $$\alpha^{\left(i\right)}=\frac{\Delta \tau^{\left(i\right)}}{\Delta \tau_{0}}$$

with $\Delta \tau^{\left(i\right)}$ being the range signal sampling rate of the *i*-th sub-swath, and $\Delta \tau_{0}$ the chosen output signal sampling rate after range scaling.

### 3.3. Azimuth Deramp

After range scaling, the standard CSA kernel [22] is adopted for range cell migration correction, range compression, second range compression (SRC), and hyperbolic phase removal. Moreover, the residual phase caused by de-rotation is compensated by:

(30) $$H_{de\_com}\left(f_{\eta }\right)=\text{exp}\left\{-j\pi \frac{f_{\eta }^{2}}{k_{rotation}}\right\}$$

However, if the azimuth inverse FFT (IFFT) is applied directly to focus the image after residual phase compensation, image folding in azimuth will likely occur. To solve the problem, a constant linear frequency modulation for the whole sub-swath is introduced, given by:

(31) $$H_{deramp}\left(f_{\eta };r_{ref}\right)=\text{exp}\left\{j\pi \frac{f_{\eta }^{2}}{k_{e}\left(r_{ref}\right)}\right\}$$

where:

(32) $$k_{e}\left(r_{ref}\right)=\frac{2v_{e}^{2}}{\lambda \left(r_{s}+r_{ref}\right)}$$

Then, after IFFT, the azimuth signal in the time domain is given by:

(33) $${S^{\prime \prime }}_{A,\eta }\left(\eta ;\eta_{a},r\right)=rect\left[\frac{\eta -\frac{r-r_{ref}}{r_{s}+r}\eta_{a}}{\frac{\lambda r_{s}}{Dv_{e}}}\right]\cdot \text{exp}\left\{-j\pi k_{e}\left(r_{ref}\right){\left(\eta -\eta_{a}\right)}^{2}\right\}$$

According to Equation (33), the deramp operation leads to a significant range-dependent time shift given by:

(34) $$\Delta T\left(r,\eta_{a}\right)=\frac{r-r_{ref}}{r_{s}+r}\eta_{a}$$

Choosing $r_{ref}$ as the middle range, the maximum value of $\Delta T\left(r,\eta_{a}\right)$ is approximately given by:

(35) $$\Delta T_{\text{max}}\approx \frac{\text{sin}\;\beta }{4\left(r_{s}+r\right)}\frac{X_{s}X_{r}}{v_{e}}$$

where β is the incident angle, $X_{s}$ and $X_{r}$ are the swath-width in azimuth and range, respectively.

**Figure 6:**
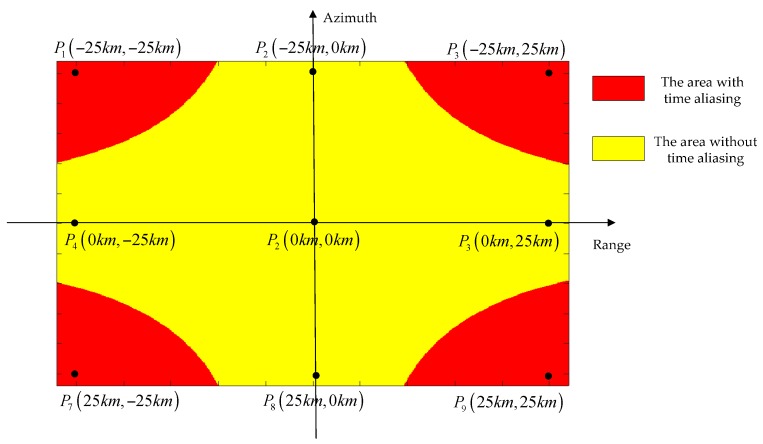
The time aliasing area.

According to Equation (35), since the time shift is proportional to the sub-swath coverage, time aliasing will occur for wide-swath coverage TOPS data processing, especially at the scene edge. With the parameters listed in Table 1, Figure 6 shows the time aliasing area, which is highlighted in red.

**Figure 7:**
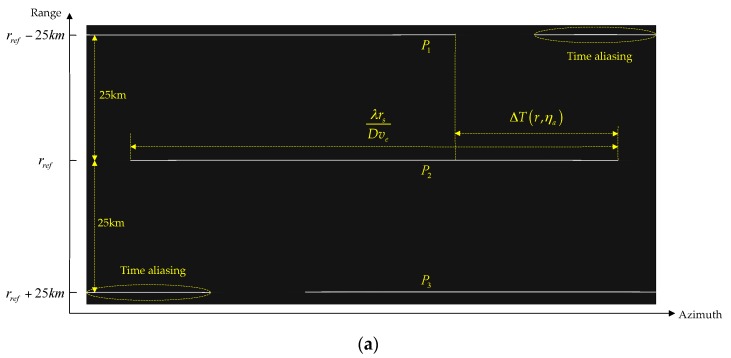

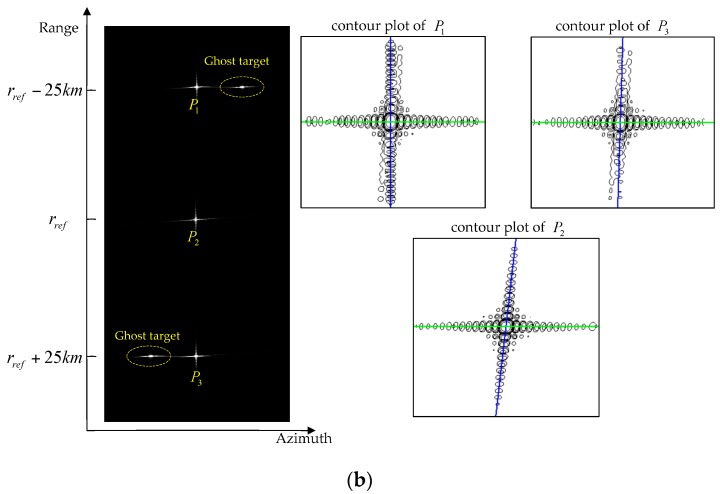
The time aliasing effects: (**a**) aliasing in the time domain after range-independent deramp; (**b**) focusing results and contour plot of the targets.

The time aliasing effects of point targets $P_{1}$, $P_{2}$ and $P_{3}$ are shown in Figure 7, with their positions indicated in Figure 6. Since $P_{1}$ and $P_{3}$ are located at the time aliasing area, significant time aliasing has occurred as shown in Figure 7a, which results in ghost appearance and contour plot distortion, with reduced azimuth resolution, as shown in Figure 7b.

### 3.4. Modified Azimuth Deramp and Chirp-z

In order to overcome the time shift problem, data division before the deramp operation can be applied, and then parameters are updated for different subdata blocks [27]. However, due to the data division, additional subdata combinations are acquired, which will lead to reduction in processing efficiency. Here, a modified method is proposed, using the range-dependent deramp function given by $H_{deramp}\left(f_{\eta },r\right)$ and:

(36) $$H_{deramp}\left(f_{\eta },r\right)=\text{exp}\left\{j\pi \frac{f_{\eta }^{2}}{k_{e}\left(r\right)}\right\}$$

With the range-dependent deramp function, we can get the time domain expression of the azimuth signal by azimuth IFFT:

(37) $${S^{\prime \prime }}_{A,\eta ,modify}\left(\eta ;\eta_{a},r\right)=rect\left[\frac{\eta }{\lambda r_{s}/Dv}\right]\cdot \text{exp}\left\{-j\pi k_{e}\left(r\right){\left(\eta -\eta_{a}\right)}^{2}\right\}$$

Compared with Equation (33), the time shift is completely compensated w.r.t. the first term. However, a range-dependent quadric phase is introduced, which should be compensated by:

(38) $$H_{scl}\left(\eta ;r\right)=\text{exp}\left\{j\pi k_{e}\left(r\right)\eta^{2}\right\}$$

By azimuth IFFT, targets are focused in the frequency domain, with position at $k_{e}\left(r\right)\eta_{a}$. Since $k_{e}\left(r\right)$ is range-dependent, azimuth geometry distortion will take place, which will be analyzed in next section.

**Figure 8:**
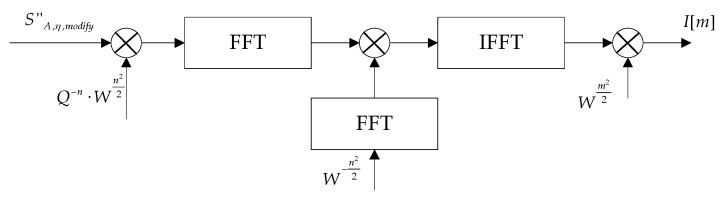
Implementation of the chirp-z transform.

As for geometry distortion, interpolations are usually performed to realize the same Doppler frequency sampling interval, resulting in additional computations. In this paper, the chirp-z transform is adopted to accommodate geometry distortion, by which more accurate geometry correction results can be obtained without interpolation. The chirp-z transform in the discrete domain is given by [28,29]:

(39) $$I[m]=CZT\left({S^{\prime \prime }}_{A,\eta ,modify}\left[n\right]\right)=W^{\frac{m^{2}}{2}}\cdot \left[\left({S^{\prime \prime }}_{A,\eta ,modify}\left[n\right]\cdot Q^{-n}\cdot W^{\frac{n^{2}}{2}}\right){⊗}_{*}W^{-\frac{n^{2}}{2}}\right]m,n\in \left[0,N_{1}-1\right]$$

where *I* is the final focused result, CZT represents the chirp-z transform, ${⊗}_{*}$ is the convolution operation which can be implemented by FFT and complex multiplications as shown in Figure 8.

(40) $$I[m]=W^{\frac{m^{2}}{2}}\cdot IFFT\left[FFT\left({S^{\prime \prime }}_{A,\eta ,modify}\left[n\right]\cdot Q^{-n}\cdot W^{\frac{n^{2}}{2}}\right)\cdot FFT\left(W^{-\frac{n^{2}}{2}}\right)\right]$$

For Q and W, they are given by:

(41) $$Q=\text{exp}\left\{-j\frac{\pi N_{1}\Delta f_{\eta }\left(r\right)}{{f^{\prime }}_{prf}}\right\},\;W=\text{exp}\left\{-j\frac{2\pi \Delta f_{\eta }\left(r\right)}{{f^{\prime }}_{prf}}\right\}$$

where $\Delta f_{\eta }\left(r\right)$ is the output frequency sampling interval, given by:

(42) $$\Delta f_{\eta }\left(r\right)=\frac{k_{e}\left(r\right)}{k_{e}\left(r_{ref}\right)}\frac{{f^{\prime }}_{prf}}{N_{1}}$$

So, the equivalent time sampling interval is:

(43) $$\Delta \eta =\frac{k_{e}\left(r\right)}{\Delta f_{\eta }\left(r\right)}=\frac{1}{k_{e}\left(r_{ref}\right)N_{1}/{f^{\prime }}_{prf}}=\frac{1}{\gamma \left(r_{ref}\right)f_{prf}}$$

Note that Δη is range-independent, which means the azimuth geometry distortion is corrected. Moreover, we can also select a fixed time sampling interval for all the sub-swathes, which avoids azimuth interpolation for the sub-swath image mosaic. 

**Figure 9:**
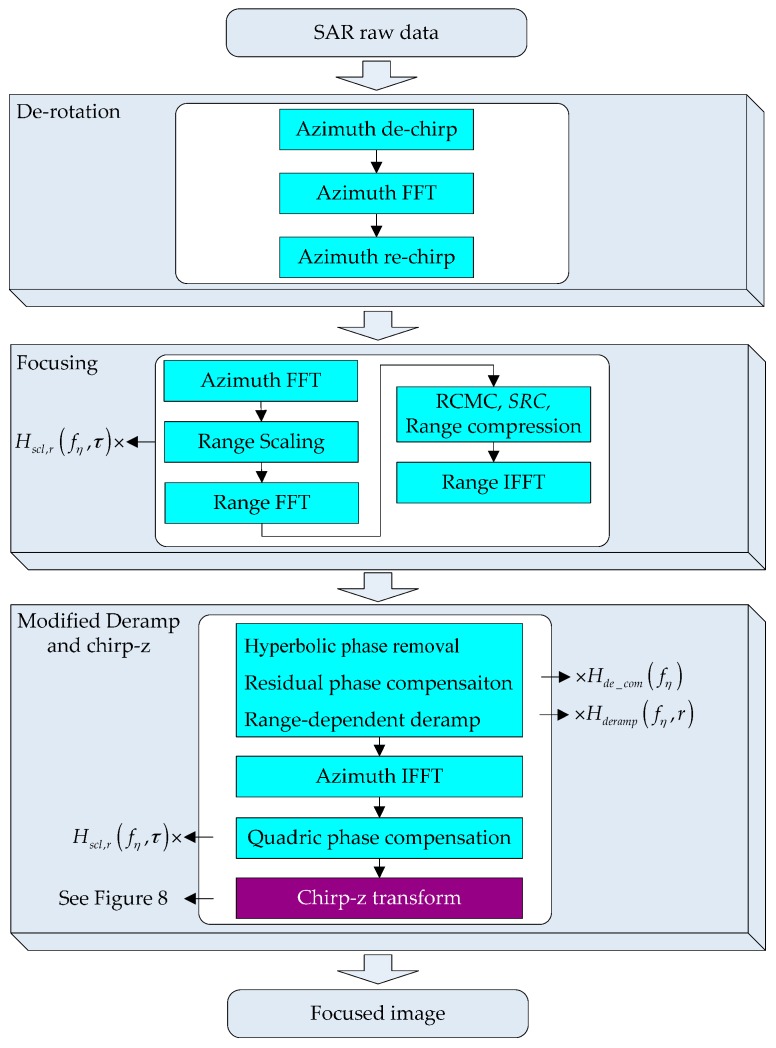
Flowchart of the proposed algorithm.

The flowchart of the proposed algorithm is given in Figure 9. First, in the derotation step, the FFT and complex multiplications are applied instead of the convolution operation in real implementation, including the azimuth dechirp, azimuth FFT and azimuth rechirp [12]. Then, after transforming the data to the range-Doppler domain using azimuth FFT, range scaling can be implemented by multiplying the data by $H_{scl,r}\left(f_{\eta },\tau \right)$. Next, transforming the data to the 2-D frequency domain by range FFT, the standard CSA kernel is employed for range cell migration correction, range compression, second range compression (SRC). After range IFFT, range compression is completed, and the data is transformed back to the range-Doppler domain, in which the hyperbolic phase removal is performed according to the standard CSA kernel. Note that the residual phase compensation and the range-dependent deramp operations are also implemented in this step by multiplying the data by $H_{de\_com}\left(f_{\eta }\right)$ and $H_{deramp}\left(f_{\eta },r\right)$, respectively. After azimuth IFFT, the data is transformed to the time domain, and we can compensate the quadric phase by multiplying the data by $H_{scl}\left(\eta ;r\right)$, which should be updated in range. Finally, the chirp-z transform is adopted to accommodate geometry distortion, which has been discussed earlier in this section.

## 4. Simulation Results and Discussion

In order to validate the proposed algorithm, the TOPS raw data of nine point targets were simulated using the parameters listed in Table 1. The nine point targets are arranged in a scene of 50 km × 50 km, as shown in Figure 6.

### 4.1. Comparative Experiments and Discussion

To show the performance of the proposed algorithm, time aliasing, geometry distortion and imaging results are disscussed with the traditional range-independent deramp algorithm and our proposed algorithm.

**Figure 10:**
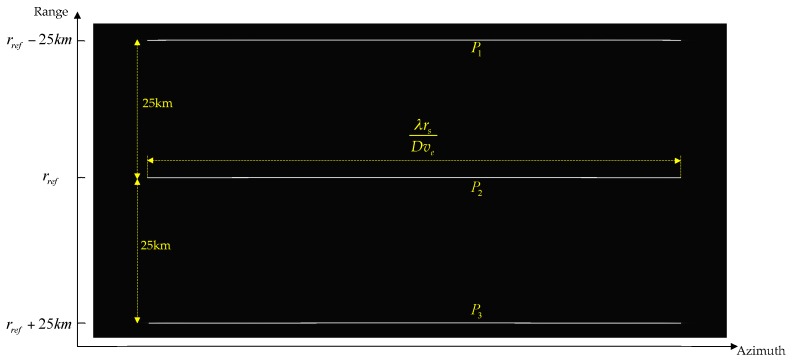
Time aliasing compensation results by adopting range-dependent deramp.

As shown in Figure 7a, using the range-independent deramp, significant time aliasing occurs, which leads to ghost target appearance and azimuth resolution reduction. By implementing the range-dependent deramp operation proposed in our algorithm, time aliasing is completely removed as shown in Figure 10.

**Figure 11:**
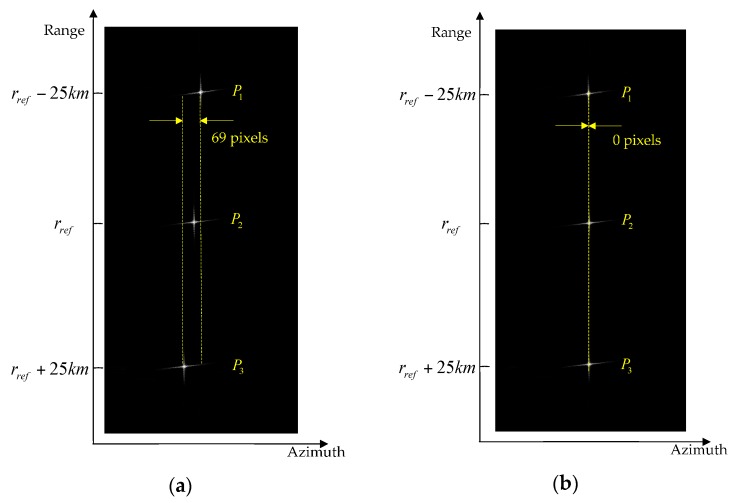
Geometry distortion correction result: (**a**) without correction; (**b**) correction by employing the chirp-z transform.

Moreover, Figure 11 illustrates the geometry correction results with the traditional FFT transform and chrip-z transform proposed in our algorithm. Due to the range-dependent deramp, the azimuth sampling rate varies with range bins. By using the FFT transform, it causes clear geometry distortions, especially at the azimuth edge, resulting in an offset of 69 pixels, as shown in Figure 11a. By employing the chirp-z transform, geometry distortions are totally compensated, as shown in Figure 11b.

**Figure 12:**
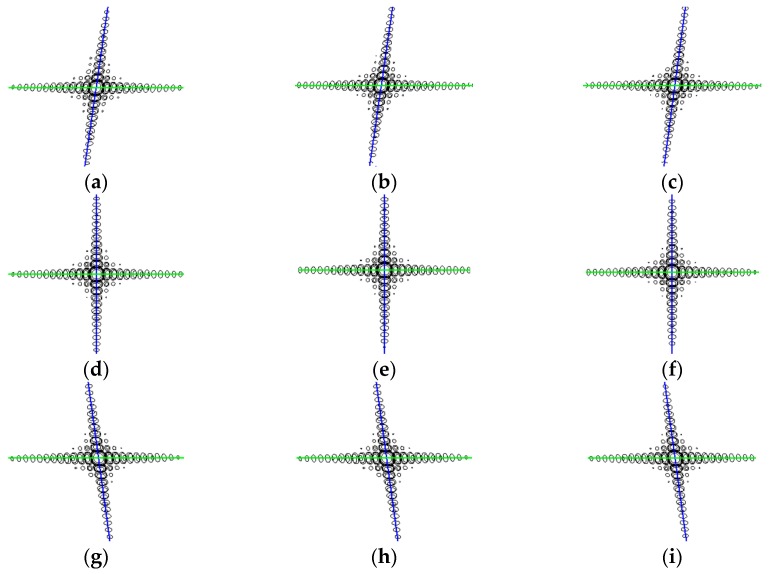
The interpolated contour plot of the nine point targets, with (**a**–**i**) corresponding to point targets from $P_{1}$ to $P_{9}$.

Finally, Table 2 shows the image results of $P_{1}$, $P_{2}$ and $P_{3}$, including the resolution, the peak sidelobe ratio (PSLR) and the integrated sidelobe ratio (ISLR). The corresponding interpolated contour plots are given in Figure 7b and Figure 12a–c, respectively. Comparing the image results, the traditional algorithm and the proposed algorithm have nearly the same resolution, PLSR and ISLR for point target $P_{2}$. However, as for $P_{1}$ and $P_{3}$, the traditional algorithm suffers from significant resolution and PSLR degradation at scene edge, while the proposed algorithm has excellent focusing performance, which means the proposed algorithm is more suitable for wide-swath TOPS data processing.

### 4.2. Imaging Simulation Experiments and Analysis

To show the focusing performance in the whole scene shown in Figure 6, the interpolated contour plot of the nine point targets and the corresponding quality measurement results are shown in Figure 12 and Table 3, respectively. All nine point targets are well focused, with no more than 1% resolution deviations compared with the theoretical values, which verifies the accuracy of the proposed algorithm. Moreover, the simulation data are processed without data division and interpolation operations by employing the proposed algorithm, which means the proposed algorithm is more efficient. Furthermore, to meet the requirement of more applications, such as high-resolution image formation, the proposed algorithm for sliding spotlight data processing will be investigated as a topic of our future study.

## 5. Conclusions

In this paper, a novel imaging algorithm has been proposed for processing wide-swath coverage spaceborne terrain observation by progressive scans (TOPS) SAR data, without raw data division and any interpolations. To overcome the Doppler frequency aliasing problem, a range-independent rotation operation was applied to compensate the Doppler bandwidth resulting from azimuth antenna-beam steering, and the azimuth signal properties after derotation were derived and discussed in detail. Then, range scaling was performed to provide the same range sampling interval for all sub-swath data, avoiding the additional range resampling operation. Moreover, a modified azimuth deramp method was presented to remove the time shift by adopting a range-dependent dermap function, which solves the time aliasing problem and avoids the appearance of ghost targets. Furthermore, to correct geometry distortions caused by the range-dependent dermap, the chirp-z transform is employed, and the same azimuth sampling interval for all sub-swath data can be obtained. As demonstrated by simulations results, the proposed imaging algorithm has performed well for the wide-swath scene. Finally, the propose algorithms also can be adopted for sliding spotlight data processing, which will be studied in future research work.

## Figures and Tables

**Table 1:** Simulation parameters.

Parameters	Value
Orbit height	630 km
Eccentricity	0.0011
Orbit inclination angle	97 deg
Argument of perigee	90 deg
Elevation	30 deg
Wavelength	0.03 m
*PRF*	5000 Hz
Antenna length	5.0 m
LFM Signal bandwidth	50.0 MHz
LFM Signal sampling rate	60.0 MHz
Azimuth swath	50 km
Range swath	50 km
Latitude of scene center	0 deg
Raw data type	64-bit complex

**Table 2:** Imaging quality analysis of comparative experiments.

Point Targets	Algorithms	Azimuth			Range		
		Resolution (m)	PSLR (dB)	ISLR (dB)	Resolution (m)	PSLR (dB)	ISLR (dB)
$P_{1}$	Traditional algorithm	16.30	−12.82	−10.15	2.71	−12.76	−9.68
	Proposed algorithm	12.33	−13.25	−10.10	2.66	−13.26	−10.11
$P_{2}$	Traditional algorithm	12.51	−13.26	−10.13	2.66	−13.13	−10.05
	Proposed algorithm	12.50	−13.25	−10.11	2.66	−13.25	−10.11
$P_{3}$	Traditional algorithm	16.39	−13.65	−11.28	2.68	−13.31	−10.00
	Proposed algorithm	12.68	−13.26	−10.12	2.66	−13.27	−10.10

**Table 3:** Quantitative analysis of the imaging results.

Point Targets	Azimuth			Range		
	Resolution(m)	PSLR (dB)	ISLR (dB)	Resolution(m)	PSLR (dB)	ISLR (dB)
$P_{1}$	12.33	−13.25	−10.10	2.66	−13.26	−10.11
$P_{2}$	12.50	−13.25	−10.11	2.66	−13.25	−10.11
$P_{3}$	12.68	−13.26	−10.12	2.66	−13.27	−10.10
$P_{4}$	12.33	−13.25	−10.10	2.66	−13.26	−10.11
$P_{5}$	12.50	−13.25	−10.11	2.66	−13.26	−10.11
$P_{6}$	12.67	−13.25	−10.12	2.66	−13.27	−10.10
$P_{7}$	12.34	−13.25	−10.11	2.66	−13.26	−10.10
$P_{8}$	12.51	−13.26	−10.12	2.66	−13.27	−10.11
$P_{9}$	12.68	−13.25	−10.10	2.66	−13.27	−10.10
